# Intergenerational Judo: Synthesising Evidence- and Eminence-Based Knowledge on Judo across Ages

**DOI:** 10.3390/sports12070177

**Published:** 2024-06-26

**Authors:** Simone Ciaccioni, Andrea Perazzetti, Angela Magnanini, Tibor Kozsla, Laura Capranica, Mojca Doupona

**Affiliations:** 1 Department of Movement, Human and Health Sciences, University of Rome “Foro Italico”, Piazza Lauro De Bosis, 15, 00135 Rome, Italy; simoneciaccioni@yahoo.it (S.C.); perazzettiandrea@gmail.com (A.P.); angela.magnanini@uniroma4.it (A.M.); 2 International Judo Federation Academy Foundation, XBX 1421 Ta’ Xbiex, Malta; tibor.kozsla@ijf.edu.mt; 3 Faculty of Sport, Physical Education Division, University of Ljubljana, Ljubljana, Aškerčeva 2, 1000 Ljubljana, Slovenia; mojca.doupona@fsp.uni-lj.si

**Keywords:** education, martial arts, combat sports, intergenerational sport, health-enhancing physical activity, wellbeing

## Abstract

In alignment with European policies regarding intergenerational sports, this study seeks to integrate existing evidence- (i.e., umbrella review) and eminence- (i.e., international validated educational courses for coaches) based knowledge on judo training across the lifespan. For evidence knowledge, searches of the Google Scholar, ISI-WoS, PubMed, and Scopus databases (PROSPERO ID: CRD42024535420) for studies focused on training youth and older judo practitioners, with no time restriction up to April 2024, were conducted. This resulted in 16 systematic reviews meeting the inclusion criteria, with an “excellent” (*n* = 6), “good” (*n* = 7), and “fair” (*n* = 3) quality according to the NIH tool. With a large sample size (*n* = 372,655), the reviews focused on wellbeing (*n* = 9; bone status, injuries, and psychophysical effects) and performance (*n* = 7; athlete success, combat time, rapid weight loss, and the Special Judo Fitness Test), examining athletic levels (novices to Olympics), sex, and age (children to older adults). For eminence knowledge, the International Judo Federation Academy courses encompassed the “Undergraduate Certificate as Judo Instructor” (UCJI), which provides a comprehensive structure for teaching judo, including techniques, moral code, roles, responsibilities, refereeing, safety, and age-specific belt examination requirements and progression, and the “Educating Judo Coaches for Older Practitioners” (EdJCO) curricula, which provide theoretical and applied modules (e.g., ageing, health, and organisation) to train older judo practitioners. The findings were summarised in a framework, highlighting the most relevant aspects of intergenerational judo programmes (i.e., aims, key characteristics, coaches’ roles, barriers, and facilitators). To develop a comprehensive educational intergenerational judo methodology, future research should examine coaches’ and practitioners’ opinions on intergenerational judo activities carried out in different national contexts.

## 1. Introduction

By combining health-enhancing physical activity for both old and young people, intergenerational sports plays a pivotal role in promoting active ageing, social inclusion, and community cohesion, with the final aim being to foster a healthier society [1]. Recently, good practices and knowledge on the benefits, barriers, and facilitators of intergenerational sports have been identified through analyses of fifteen initiatives across the European Member States [1]. The European Union indicates the opportunity for projects involving young people and the elderly in “forms of co-learning aimed at enhancing the resources of both and opening up the possibility of better mutual understanding” [2]. Moreover, “a good intergenerational relationship represents one of the conditions for finding a purpose in the third and fourth age not only in terms of assistance and/or therapy, but also a purpose useful to the younger generations, still open to the future, rich in hope and desire for life” [3]. The goal is to strive for a balanced intergenerational relationship that combines autonomy with solidarity, recognising that each generation not only inherits from the past but also has much to learn from the generations that follow. This happens even more so when it comes to experiential intergenerational learning, as is the case in sports practices. In this case, knowledge is generated through the reworking and transformation of experience, which can be supported and guided by the different ages involved [4].

Interaction with another person in a specific relationship and with others in the context of group dynamics (which often alternate and coexist in non-formal environments involved in the project) promotes participatory movement and moments, as well as providing possibilities of discovering differences, histories, and languages, going deep, and stimulating a greater degree of awareness of one’s own identity and the group identity. Constructing proximity practices makes “the other” near, visible, and recognisable, and it reduces the temptation to flee and to distance oneself from the differences, which are actual or perceived as such because of a stereotype [5]. In this direction, Kuehne [5] emphasises that social identity theory suggests that, to avoid stereotypical behaviour among intergenerational programme participants, it is important to focus less on the age difference between participants (and, thus, stereotypes) and more on the individual qualities of participants, regardless of their group differences (e.g., age). This theory seems especially appropriate for intergenerational programmes that are based primarily on the mutual interests of the participants [5].

Intergenerational sports activities, grounded in the concept of co-learning, mutual intergenerational contact, and exchange, can promote the idea of active citizenship, which ensures that every participant is fully aware of working together for the common good [6]. Furthermore, activity theory has also been applied to older adults’ involvement in intergenerational programmes. This theory suggests that, as ageing adults lose various social roles in society, they maximise their sense of wellbeing, life satisfaction, and self-concept when the lost roles are replaced with new ones [7]. Intergenerational programme participation has clearly been viewed as a potential “new” role for older adults that can contribute towards “successful ageing” and improved wellbeing [8]. Intergenerational programmes have been advocated as a means of promoting health and wellbeing among both the young and the old [9]. Kaplan [10] argues that the implementation of intergenerational programmes contributes to personal growth and the development of social skills, as they are “social vehicles that create purposeful and ongoing exchange of resources and learning. In a nutshell, it is about intergenerational engagement—the full range of ways in which young people and older adults interact, support, and provide care for one another”. Hatton-Yeo and Ohsako (2000) [11], Ellis and Granville (1999) [12], and Rosebrook (2002) [13] emphasise reciprocity as an indispensable characteristic in intergenerational learning programmes (tutoring and mentoring) aimed at the real sharing and building of knowledge to achieve better coexistence and—as highlighted by Toffano Martini and Zanato Orlandini (2012)—the reconstruction of “that solidarity among generations that, appreciating every age, does not lose any” [2].

Despite researchers’ and practitioners’ growing interest, the exploration of intergenerational sport is a relatively uncharted territory that warrants a comprehensive investigation [14,15]. Considering their focus on self-discipline, balance, the mind–body connection, and adaptability to different populations, martial arts offer a unique blend of physical and mental health, making them well suited as physical activities fostering solidarity and holistic wellbeing in both younger and older generations [9,16,17,18]. As a modern martial art and inclusive combat sport created in Japan in 1882 by Jigoro Kano, judo (jū: gentle; dō: way) has been recognised for its multifaceted nature, including physical and mental benefits [19,20,21,22,23]. Judo promotes physical efficiency and mutual respect through gymnastics (i.e., taiku), free practices (i.e., randori), prearranged techniques (i.e., kata), and combats (i.e., shiai) [24,25]. Its multimodal practice can be adjusted to the physical capabilities of the practitioners, having psychological and social effects on overall wellbeing at different stages of the life course [26,27,28,29,30]. Furthermore, judo has inherently had an educational foundation since its genesis and development, which fosters a transfer of knowledge and skills from one generation to another and enhances the learning experience, accompanying its practitioners on the path of knowledge and awareness and in developing a sense of community [31,32,33,34]. According to Kano, the harmony and balance achieved by individuals cannot remain confined to the gymnasium and must serve as the basis for active social participation, involving individuals’ constant commitment to contributing to social change [35]. Thus, the aim of judo is to provide conceptual, physical, cognitive, and moral tools so that individuals can navigate their own lives and contribute to the growth of humanity [25,35]. Within this framework and under the banner of an education perspective that persists throughout an individual’s lifespan, several works in the literature have emphasised the value of intergenerational activities, despite there still being little conceptual clarity on the matter [35]. Despite a solid literature highlighting the outcomes of judo training across the lifespan [36,37,38,39], there is a lack of information on certified programmes for intergenerational groups.

In fact, worldwide judo coach education typically encompasses a combination of formal (i.e., academic or university-based courses), non-formal (i.e., planned programmes offered by associations and federations), and informal (i.e., practical experiences and theoretical learning within and outside judo halls—dojo) instruction [38]. The International Judo Federation (IJF) and national governing bodies often oversee coach education. Currently, key courses include (i) IJF Academy programmes for judo instructors, coaches, and managers, which offer different levels of certification and cover a wide range of judo-related technical, tactical, physical, psychological, and organisational aspects across the lifespan [38,39,40,41,42,43]; (ii) national coaching certification programmes, aligned with the sport structures of individual countries and covering coaching principles, sport-specific skills, and athlete development [44]; (iii) university courses offering degrees or programmes in sports coaching, some of which include specific modules on judo coaching (e.g., sports science, coaching theory, and practical coaching experience) [1]; and (iv) specialised workshops and seminars, conducted by experienced judo practitioners, focusing on specific aspects such as competition preparation, technical skills, and athlete psychology [1].

To design effective and safe intergenerational judo programmes, it is essential to gather extensive theoretical knowledge and applied information on the physical, mental, and social impacts of judo training across generations. Therefore, the main purpose of this study was to develop a framework for intergenerational judo by summarising the knowledge on the effects of judo training through a systematic literature review following international guidelines [45] and a content analysis of the educational curricula for coaches of the International Judo Federation Academy [40].

## 2. Methods

### 2.1. Design

This study is part of the co-funded European Erasmus + Sport “Judo connecting Older and Younger generations” project (JOY, ERASMUS-SPORT-2023-SCP, number: 101133628). The research encompasses evidence- (i.e., umbrella review) and eminence- (i.e., online international educational courses for judo coaches)based knowledge.

### 2.2. Protocol and Registration

Registered on PROSPERO (ID: CRD42024535420), the present umbrella literature review of systematic reviews was based on the Preferred Reporting Items for Systematic Reviews and Meta-Analyses (PRISMA) and Preferred Reporting Items for Overviews of Reviews (PRIOR) guidelines [45,46].

### 2.3. Eligibility (i.e., Inclusion and Exclusion) Criteria

The inclusion criteria for the selection of studies encompassed the following: (i) original peer-reviewed review articles published without a time restriction up to April 2024 in the English language; (ii) studies involving practitioners within the following age groups: children (i.e., 3–12 years), adolescents (i.e., 13–17 years), young adults (i.e., 18–24 years), middle-aged adults (i.e., ≥45–65 years), and older adults (i.e., ≥65 years) [47]; (iii) studies with a literature review design (e.g., systematic reviews and meta-analyses); (iv) effects of or associations with judo training; and (v) outcomes on the following research areas: health/wellbeing (e.g., cognition, injuries, safety, and weight loss) and performance (e.g., nutrition, skills, techniques, and tests). When relevantly connected to the previous research areas, other contents were also considered, such as those of social sciences (e.g., history-, philosophy-, and theory-related aspects). Conversely, the exclusion criteria encompassed the following: (i) focus on the effects of or main exposure to other sports; (ii) other study designs (e.g., longitudinal, experimental, cross-sectional, and case studies), including non-systematic reviews (e.g., scoping, narrative, and brief); and (iii) non-peer-reviewed publications (e.g., letters to the editor, translations, and book reviews).

### 2.4. Information Search and Study Selection Process

In April 2024, a systematic literature search of review articles was performed on the Google Scholar, Institute for Scientific Information Web of Science (ISI WoS), PubMed (National Library of Medicine), and Scopus databases. The used search string was (judo) OR (judoka*) OR (judoist*) OR (martial AND art*) OR (combat AND sport*) AND (adolescen*) OR (child*) OR (development) OR (growth) OR (juvenile) OR (minor) OR (p?ediatric) OR (“school age*”) OR (teen*) OR (youth) OR (“young people”) AND (adult*) OR (ag?ing) OR (aged) OR (elder*) OR (master*) OR (old*) OR (senior*) OR (veteran*) AND (andragogy) OR (anthropolog*) OR (attitude) OR (behavior*) OR (communication*) OR (culture) OR (discipline*) OR (education) OR (emotion*) OR (enjoyment) OR (happiness) OR (knowledge) OR (interaction) OR (interpersonal AND relations) OR (internet) OR (mental AND health) OR (mental AND processes) OR (negotiating) OR (performance) (personality) OR (pleasure) OR (respect) OR (satisfaction) OR (self-concept) OR (self-control) OR (self-esteem) OR (sociology) OR (social AND participation) OR (social AND skills) OR (test*) OR (values). The asterisks (*) and the question marks (?) were used to pull all derivations of similar root words (i.e., old adult * = old adult and old adults) and for single character searching (e.g., ag?ing = ageing or aging), respectively. To ensure the inclusion of the most updated reviews, alert notifications for new publications were activated until May 2024. To ensure conformity with the inclusion criteria, two authors with a PhD in sports sciences, one of whom is a judo coach and has a 4th dan black belt, independently completed the systematic review phases (i.e., the screening, quality assessment, and data extraction of pertinent studies). When disagreements arose regarding eligibility, a third author’s perspective was sought. The studies retrieved underwent screening based on their titles, abstracts, and full texts. To ensure a comprehensive search for relevant articles, the snowball technique was employed.

### 2.5. Data Extraction, Analysis, and Synthesis

The data extraction of the final review sample was based on the PRIOR approach [46]. When available, the information extrapolated and examined included the author(s); publication year; journal; country in which the leading author conducted the study; research area; review aim; sample number, age category, mean and standard deviation, range, sex, and sport level of judo practitioners; main topic; and outcomes. According to the PRISMA guidelines [45], data were processed through a qualitative synthesis. The effects and correlates of judo training were considered in relation to the participants’ chronological age and according to the research area (i.e., wellbeing and performance). Then, detailed tables and figures reporting the major characteristics and findings of the selected studies were created.

### 2.6. Quality Assessment of Systematic Literature Reviews

To assess the methodological quality, the National Institute of Health (NIH; https://www.nhlbi.nih.gov/health-topics/study-quality-assessment-tools (accessed on 13 May 2024)) tool was used, which consists of 8 items that appraise the following issues: (i) focused research question; (ii) eligibility (i.e., inclusion and exclusion) criteria; (iii) comprehensive and systematic approach used for literature search; (iv) dual review used for determining which studies to include and exclude; (v) quality for internal validity; (vi) list and description of included studies; (vii) publication bias; and (viii) heterogeneity (e.g., clinical, methodological, and statistical). Thus, an “excellent” (7–8 pt.), “good” (5–6 pt.), “fair” (4–3 pt.), or “poor” (2–0 pt.) judgment was assigned to each included manuscript based on the NIH results (Table 1 and Supplementary Material S1).

### 2.7. Online Educational Courses for Judo Coaches

In considering the IJF policy to certify coaches, the following IJF Academy educational programmes were analysed: (i) the Undergraduate Certificate as Judo Instructor—UCJI course (https://academy.ijf.org/student-handbook (accessed on 13 May 2024)), including 14 theoretical (e.g., classification, culture, health, history, organisation) and practical (e.g., coaching role, long-term athlete development, rules, and techniques) modules for judo instructors and coaches working with youth and beginner athletes, and (ii) the Educating Judo Coaches For Older Practitioners—EdJCO course (https://edjco.eu/ (accessed on 13 May 2024)), encompassing 6 (e.g., organisation and environment, ageing, safety, physiology, psychology, and teaching and training) modules.

### 2.8. Analysis and Quality Assessment of Online Educational Courses for Judo Coaches

To analyse and assess the quality and effectiveness of the international judo online courses, the adapted form of the National Standards for Quality Online Learning (NSQOL) tool [48,49] was used, and it consists of the following five criteria: (A) Course Overview and Support (six items: the syllabus, minimum skills and technology requirements, information on how to communicate with the instructor, course expectations and policies, grading policies and practices, and technical support); (B) Content (six items: objectives, digital literacy, supplemental learning resources, culturally diverse perspectives, accuracy, and currency of materials); (C) Instructional Design (eight items: activities for learning ownership, self-monitoring, and promotion; logical sequence of units and lessons; appropriateness of content; introductory activities; variety of paths; learner–instructor interaction; effectiveness; and engagement of materials); (D) Learner Assessment (five items: varied levels of proficiency, valid assessment measures, unit–learner assessment link, self-monitoring and reflection routine, and flexibility in mastery demonstration); (E) Accessibility and Usability (four items: logical, consistent, and efficient navigation; readability; accessibility; and facility use); (F) Technology (five items: privacy and confidentiality, tool support, activity adaptability, content release control, and score and record functionality); and (G) Course Evaluation (three items: course effectiveness, reviewing, and continuous improvement). Each course was rated on the extent to which it meets the criteria, with 1 pt., 2 pt., and 3 pt. indicating that a course does not meet, partially meets, and fully meets the criteria, respectively. This evaluation is key to ensuring that online courses provide students with access to quality instruction and resources. Thus, a “good” (87–111 pt.), “fair” (62–86 pt.), or “poor” (37–61 pt.) rating was assigned to each included course based on the NSQOL results.

**Table 1:** First author and publication year, country, research area, journal, and quality assessment of the systematic literature reviews included in this umbrella review.

Author (Year)	Country ^a^	Research Area	Journal ^b^	Quality Assessment	
				Score	Evaluation Rating
Barreto et al. (2024) [50]	Brazil	Performance	*IDO*	7/8	Excellent
Barreto et al. (2022) [51]	Brazil	Performance	*Front Psychol*	8/8	Excellent
Ciaccioni et al. (2019) [52]	Italy	Wellbeing	*JSCR*	7/8	Excellent
Gutierrez-Garcia et al. (2018) [53]	Spain	Wellbeing	*IDO*	5/8	Good
Hlasho et al. (2023) [54]	South Africa	Performance	*Heliyon*	4/8	Fair
Lakicevic et al. (2020) [55]	Italy	Wellbeing	*Nutrients*	7/8	Excellent
Lakicevic et al. (2024) [56]	Italy	Wellbeing	*ERAP*	5/8	Good
Lockhart et al. (2022) [57]	UK	Wellbeing	*IJERPH*	7/8	Excellent
Mooren et al. (2023) [58]	Netherlands	Wellbeing	*TSM*	8/8	Excellent
Palumbo et al. (2023) [43]	Italy	Wellbeing	*Sports*	6/8	Good
Pečnikar et al. (2020) [59]	Slovenia	Wellbeing	*AoB*	5/8	Good
Pocecco et al. (2013) [60]	Austria	Wellbeing	*BJSM*	3/8	Fair
Rossi et al. (2022) [61]	Italy	Performance	*IJERPH*	6/8	Good
Schoof et al. (2024) [62]	Netherlands	Performance	*IJSSC*	6/8	Good
Sterkowicz et al. (2019) [63]	Poland	Performance	*Sports*	5/8	Good
Sterkowicz et al. (2014) [64]	Poland	Performance	*JSCR*	3/8	Fair

^a^ Country: first author affiliation. ^b^ Journals: *AoB* = *Archives of Budo*, *BJSM* = *British Journal of Sports Medicine*, *ERAP* = *European Review of Applied Psychology*, *Front Psychol* = *Frontiers in Psychology*, *IDO* = *Ido Movement for Culture Journal of Martial Arts Anthropology*, *IJERPH = International Journal of Environmental Research and Public Health*, *IJSSC* = *International Journal of Sports Science & Coaching*, *JSCR* = *Journal of Strength and Conditioning Research*, *TSM* = *Translational Sports Medicine*. Quality evaluation rating: excellent = 7–8, good = 6–5, fair = 4–3, poor = 0–2 (https://www.nhlbi.nih.gov/health-topics/study-quality-assessment-tools (accessed on 13 May 2024)).

## 3. Results

### 3.1. Study Selection and Data Collection

**Figure 1:**
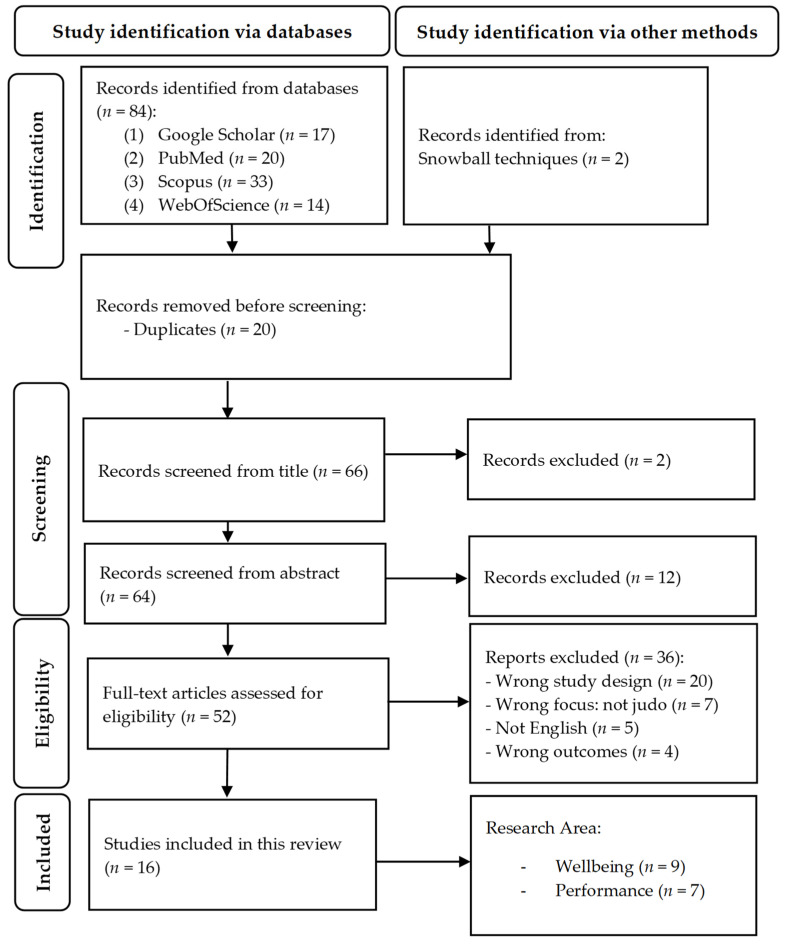
Flowchart of the systematic process of review (*n* = number).

The PRISMA flow diagram (Figure 1) shows the article selection process based on the title, the abstract, and the full text, reporting the reasons for exclusion. The electronic search strategy identified 84 records, with 14 articles retained after the screening of the title, abstract, and full text, further implemented with 2 additional contributions identified through the snowball technique. The final list of 16 included manuscripts is shown in Table 1 and Table 2, ordered alphabetically according to their reference (i.e., authors’ name order).

### 3.2. Review Characteristics

Considering the leading author affiliation, Table 1 highlights that most of the studies (*n* = 13/16) were developed in Europe, whereas a lower geographical representation was found for South America (*n* = 2/16) and Africa (*n* = 1/16). Actually, most of the studies showed international [43,56,57,59,61] or intercontinental [50,51,54,55,60,63,64] collaborations. From the first systematic literature review focusing on the epidemiological aspects of injuries in judo published in 2013, the use of this study design to investigate judo training for wellbeing (*n* = 9) or performance (*n* = 7) rose (2012–2019: *n* = 5 reviews, 2020–2023: *n* = 8 studies), with three reviews already published this year (2024). Regarding the editorial aspects, *Ido Movement for Culture Journal of Martial Arts Anthropology* (Stowarzyszenie Idokan Polska, Strzyżów, Poland), *International Journal of Environmental Research and Public Health* (Multidisciplinary Digital Publishing Institute, Basel, Switzerland), *Journal of Strength and Conditioning Research* (National Strength and Conditioning Association, Colorado Springs, CO, USA), and *Sports* (Multidisciplinary Digital Publishing Institute, Basel, Switzerland) published two studies each. Conversely, the following six journals published one review only: *Archives of Budo* (Archives of Budo Bartlomiej Barczynski, Warsaw, Poland), *British Journal of Sports Medicine* (BMJ Publishing Group, London, UK), *European Review of Applied Psychology* (Elsevier Masson SAS, Toulouse, France), *Frontiers in Psychology* (Frontiers Media SA, Lausanne, Switzerland), *International Journal of Sports Science & Coaching* (SAGE Publications Inc., London, UK), *Nutrients* (Multidisciplinary Digital Publishing Institute, Basel, Switzerland), and *Translational Sports Medicine* (Wiley-Hindawi, London, UK).

### 3.3. Judo Practitioners, Topics, and Results

Overall, the 16 systematic literature reviews had a large sample size (*n* = 372,655), including children, adolescents, and young to older adults (Table 2). Whilst two studies addressed aspects related to one generation [53,55], one theoretical study [54] focused on general aspects that could be applied to both individuals and organisational bodies (e.g., federations). The majority of the studies [43,50,51,52,53,56,57,61,63,64] included a high representation of male judoka (≈62%), whereas six reviews [54,55,58,59,60,62] did not report this information. Only 2 studies [53,54] did not specify the athletic level of the considered judoka, and the remaining 14 reviews encompassed novice, recreational, amateur, and competitive (regional to world-class elite) athletes.

Regarding the topics, two main areas emerged: nine reviews (56%) focused on wellbeing-related aspects (i.e., bone health/status, injury epidemiology and prevention, and the psycho-physical effects of judo training), and the remaining seven studies (44%) addressed performance-related themes, including athletic success, combat time, rapid weight loss, and performance parameters such as normative values for the Special Judo Fitness Test.

Finally, the 16 reviews highlighted the following results: 1. Rule changes impact both male and female judo competition trends, leading to homogeneity by weight divisions and increased Golden Score (i.e., match winning for the first judoka who score ippon or wazari after regular time) occurrences [50,51]. 2. Judo impacts bones: site-specific BMD accrual and reduced bone impact force during falls [52]. 3. Young judoka show improved fitness but also higher anger levels [53]. 4. Professionalism and clear long-term plans enhance judo athlete performance and participation [54]. 5. Rapid weight loss affects mood (e.g., tension, anger, and fatigue), with an unclear impact on performance [55,56]. 6. Ukemi techniques prevent head and neck injuries [57]. 7. Whilst injury risks in judo involve sprains, strains, contusions, and rare brain/spine injuries [60], judo tournament injuries vary, with common locations being the head, hand, knee, elbow, and shoulder [58]. 8. Judo training later in life has positive effects on health, functional fitness, and psychosocial aspects [43]. 9. Judo benefits people with special conditions, focusing on quality of life, motor skills, and health promotion [59]. 10. Emotional responses in judo vary based on performance outcomes [61]; physiological characteristics are essential for managing judo combats [62]. 11. Age-related differences highlight the importance of throws in judo performance, with senior athletes performing better in terms of throws and post-Special Judo Fitness Test heart rate [63,64].

### 3.4. Study Quality of the Included Reviews

Table 1 provides a summary of the quality assessment (i.e., excellent = 7–8 pt., good = 6–5 pt., fair = 4–3 pt., and poor = 0–2 pt.) of the included studies, which were evaluated in detail based on the eight specific items of the NIH tool used to test for potential flaws in study methods and the implementation of systematic reviews and meta-analyses (as reported in Supplementary Material S1). Whilst all studies applied a literature search strategy using a comprehensive and systematic approach, most of them (*n* = 15/16) presented (i) a focused question, adequately formulated and described, and (ii) predefined and specified eligibility criteria for included and excluded studies. Conversely, only two reviews [54,60] did not clearly or systematically list the important characteristics and results of each of the included studies. Twelve reviews clearly reported that titles, abstracts, and full-text articles were dually and independently reviewed for inclusion and exclusion to minimise bias; in a slightly lower number of reviews (*n* = 11), the quality of each included study was rated independently by two or more reviewers using a standard method to appraise its internal validity. Finally, seven reviews quantitatively or qualitatively assessed the experimental or methodological heterogeneity, whereas two studies only assessed and clearly described the likelihood of publication biases.

### 3.5. Quality of Online Judo Courses

The online courses “Educating Judo Coaches for Older Practitioners—EdJCO” and the “Undergraduate Certificate as Judo Instructor—UCJI” achieved, respectively, a total score of 87 out of 111 and 96 out of 111 in the NSQOL tool, thus being classified as “good” (Table 3 and Supplementary Material S2). According to Section A—“Course Overview and Support” (EdJCO = 15/18—83.3% vs. UCJI = 14/18—77.8%)—both courses offer a detailed overview and syllabus, ensuring clarity for learners, providing guidance on communicating with the instructors, and establishing clear policies aligned with learning expectations (as well as providing adequate learning resources and materials to students prior to the course start). For Section B—“Content” (EdJCO = 16/18—88.9% vs. UCJI = 18/18—100%)—the courses ensure that its objectives are measurable and clearly outlined, defining what learners will achieve upon successful completion. Expectations are aligned with course objectives, reflecting the course’s structure, and cultural diversity is reflected in the course materials, which are accurate and updated, adhering to content standards. Similarly, for Section C—“Instructional Design” (EdJCO = 19/24—79.2% vs. UCJI = 24/24—100%)—the courses utilise several instructional materials and activities to engage learners and facilitate the achievement of learning objectives, organised in a logical sequence of units and lessons. Furthermore, in this section, it was voted that the course encourages learner and instructor interactions, offering feedback opportunities on learner progress, presenting content through instructional materials and resources. According to Section D—“Learner Assessment” (EdJCO = 11/15—73.3% vs UCJI = 13/15—86.7%)—the courses employ various assessment tests to evaluate students’ understanding, including self-check and practice assignments that offer immediate feedback and opportunities for revision. However, the assessment materials may lack flexibility in allowing learners to demonstrate mastery through a variety of test approaches. In fact, in these online courses, the assessment process focuses only on providing feedback on correct and incorrect responses, without assigning any grade. Regarding Section E—“Accessibility and Usability” (EdJCO = 12/12—100% vs. UCJI = 12/12—100%)—both courses’ designs ensure accessibility, offering materials and activities that suit learners’ needs without requiring specific technological skills. Multimedia elements enhance the courses’ usability and readability for all participants. In the same way, Section G—“Course Evaluation” (EdJCO = 7/9—77.8% vs. UCJI = 7/9—77.8%)—highlights how the courses are regularly reviewed to maintain up-to-date content, integrating feedback from ongoing evaluations. On the contrary, according to Section F—“Technology” (EdJCO = 7/15—46,7% vs. UCJI = 8/15—53.3%)—the courses may require further improvements in options for instructors to tailor activities to accommodate learners’ needs and preferences and to score, record assessments, and calculate earned course points or grades.

### 3.6. Novel Framework for Intergenerational Judo Activities including Recommended Online Courses for Judo Coaches

**Figure 2:**
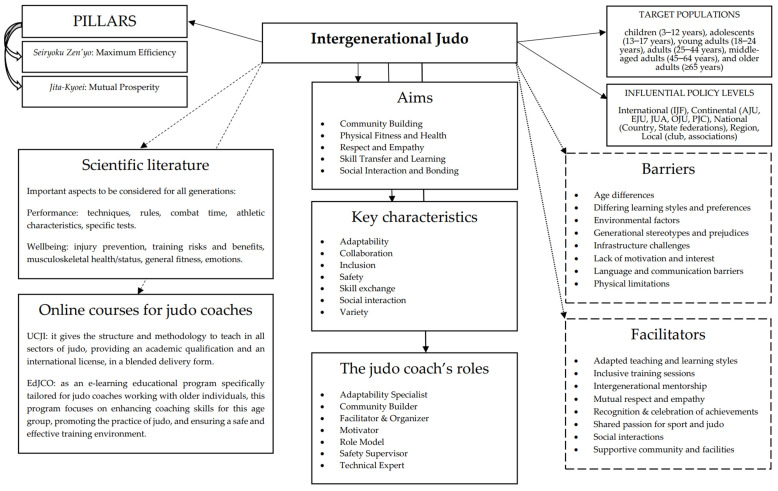
Overview of guidelines for intergenerational judo activities based on the judo fundamentals [25], systematic literature reviews [43,50,51,52,53,54,55,56,57,58,59,60,61,62,63,64], and online judo courses contents and methods [38,40]. Note: AJU = African Judo Union, EdJCO = Educating Judo Coaches for Older practitioners; EJU = European Judo Union, IJF = International Judo Federation; JUA = Judo Union of Asia, OJU = Oceania Judo Union, PJC = Pan-American Judo Confederation; UCJI = Undergraduate Certificate as Judo Instructor.

An overview of the guidelines for intergenerational judo activities is shown in Figure 2. The two main principles of judo, i.e., maximum efficiency (in Japanese “Seiryoku-Zenyo”,精力善用) and mutual prosperity (“Jita-Kyoei”, 自他共栄), support the whole framework [25]. Whilst wellbeing and performance are the two main characteristics currently studied in the systematic literature reviews on judo training [43,50,51,52,53,54,55,56,57,58,59,60,61,62,63,64], the International Judo Federation Academy operates two online judo courses for coaches interested in developing teaching and training skills for all judo sectors and ages [40]. By means of intergenerational activities and interactions, judo can foster a positive social transformation based on respect- and empathy-building among participants. Both the literature and the judo courses highlight the following crucial aspects: (i) inclusivity (i.e., everyone can participate); (ii) the prioritisation of safety in all activities; (iii) adaptability, tailoring programmes to connect different age groups; (iv) social exchange, facilitating meaningful interactions; and (v) community engagement, encouraging involvement by means of free games and structured activities. The judo coach plays several roles, acting as not only a technical expert but also a mentor guiding and inspiring participants, as well as a role model demonstrating positive behaviours while ensuring safe practice and building solid connections. Finally, to overcome individual and social barriers (e.g., age, environment, and language limits) and benefit from several facilitators (e.g., passion, education, and support), the judo intergenerational activities need to be enacted at various levels starting from the international and national federations up to the local contexts.

## 4. Discussion

To develop a framework for intergenerational judo, this study aimed to gain a comprehensive understanding of judo’s impact across age groups by synthesising evidence- and eminence-based knowledge and bridging theoretical (systematic reviews’ best research evidence) and applied (IJF Academy customised and global curricula) information. Overall, the 16 included studies had an “excellent” (*n* = 6), “good” (*n* = 7), and “fair” (*n* = 3) quality, with a score ≥ 87 pt. The selected UCJI and EdJCO international online courses were both evaluated positively.

Intergenerational connections are a powerful force that transcend time, age, and experience [1]. In the world of sports, judo—rooted in discipline, respect, and balance—offers a unique blend of physical prowess and mental acuity [25]. By means of intergenerational activities, it can foster wellbeing, mutual understanding, and a healthier society [38]. Born at the end of the XIX century in a period of strong societal changes in Japan, judo was developed by Professor Jigoro Kano as an existential educational discipline [25,35]. Because of its multifaceted nature, judo can be considered a tool of (i) physical education, where techniques—both in structured (i.e., kata) and free (i.e., randori) practice—build physical competence [31,32,33]; (ii) cognitive achievement, as practitioners learn to read opponents and respond effectively, maturing mental agility, strategic thinking, and adaptability [16,61,65,66,67]; and (iii) social and moral development, since its values of “gentleness” (jū) and mutual welfare and benefit (jita kyōei) guide behaviours both on and off the mat [18,43,53,68,69].

This umbrella review synthesised 16 systematic literature reviews published in the last 10 years (2013–2024), drawing insights from a vast sample size (*n* = 372,655). From children to older adults, judo’s impact reverberates through time. Regarding the gender dimension, male judoka still dominate the representation (≈62%) in the majority of studies, mirroring some stereotypes and discrimination, which were part of the Japanese culture and struggled to disappear [69]. In fact, as Miarka et al. (2011) stated, “There are still traces of Japanese resistance to changes in the role of women in judo, exemplified by the late promotion of Keiko Fukuda to 9th dan and the obstacles for women to act as international referees. However, judo itself has evolved, facing the challenges of universalisation and sportisation in new historical contexts”. The studies also highlighted the considerable variety in judo athletic levels: from novice recreational practitioners playing judo for leisure reasons to competitive beginners and elite athletes, the literature seems to echo the diversity of the judo population around the globe [19]. Nine reviews delved into wellbeing-related themes, particularly bone health and injury epidemiology and prevention, as well as the psycho-physical effects of judo training. Conversely, the remaining seven studies focused on performance, considering hot topics such as rule changes, athlete success, combat time, rapid weight loss, and judo-specific tests (e.g., the Special Judo Fitness Test).

Kuehne (2003) [5] suggests focusing less on age differences and stereotypes and more on individual qualities when developing programmes for both young and older practitioners, as intergenerational judo is not just about physical throws; it is about bridging knowledge gaps and fostering a healthier, more connected society [38,70,71]. Social identity theory supports this approach, especially in programmes where participants share mutual interests [5]. When young and old persons engage in sports together, they defy stereotypes. The focus shifts from age to shared passions [9]. Thus, judo dojo become spaces where wisdom and energy collide, transcending generational boundaries [38]. If older adults lose their social roles, intergenerational programmes can offer a fresh purpose—a chance to contribute to successful ageing and improved wellbeing [7,8,70]. In particular, judo can become more than physical exercise; it becomes a lifeline where stress dissipates, camaraderie blooms, and mental clarity reigns.

In the dojo, the beginner learns from the experienced judoka, while the exchange of knowledge and skills enriches both. Older practitioners pass down wisdom, while younger ones infuse energy and enthusiasm. Mutual respect binds them together. Judo’s ethos revolves around respect for oneself and others. Regardless of age, everyone bows to their partner before practice—a gesture of mutual acknowledgment [25,34,35]. Indeed, judo presents physical challenges at every stage of life [53,71,72]. Whether mastering a new throw or refining a technique, practitioners find fulfilment in progress [31,32,33]. The young learn discipline, teamwork, and resilience; the older practitioners stay agile, sharp, and socially engaged [30,53,73,74]. As both physically and metaphorically they gracefully fall, judo counters isolation and provides purpose [25,61]. As reciprocity defines intergenerational learning, programmes that emphasise tutoring, mentoring, and shared knowledge create bonds [11,12]. Thus, through judo, solidarity can be reconstructed, valuing individuals of all ages without neglecting any [35]. As Kano envisioned, judo’s harmony extends far beyond the mat, enriching lives and contributing to a better world [25,35,69].

To develop, lead, and revise inclusive and dynamic intergenerational courses, a crucial role is played by the judo coach, who applies both pedagogical and andragogical approaches to accommodate diverse age groups, focusing on safety, physiology, and psychology [38]. Promoting and facilitating lifelong learning, a continuous, self-motivated pursuit of knowledge and skills, the judo coach adapts teaching methods to address the unique needs of older and younger practitioners, ensuring a supportive environment that fosters physical and mental wellbeing across generations [1,39]. Useful resources support the personal and professional development of the judo coach. In particular, the UCJI online course provides comprehensive training to instructors for youth and novice athletes, integrating theoretical knowledge with practical coaching and judo techniques while promoting continuous education, whereas the EdJCO course aims to equips judo coaches with the necessary knowledge, skills, and attitudes to effectively teach and train older practitioners.

In providing a solid foundation of evidence-based insights into intergenerational judo through a systematic umbrella review [45,46] and the analysis of two validated online international courses for judo coaches, some practical advice on how to successfully implement judo techniques and instructional strategies in intergenerational contexts emerged [38,40]. By employing this study’s information (i.e., age-specific pedagogy and andragogy, adaptive teaching methods, progressive skill development, intergenerational activity organisation, and performance- and wellbeing-related aspects), judo coaches can create a more inclusive, effective, and enriching training environment that caters to the unique needs of practitioners at different stages of their life. Moreover, these innovative resources promote a holistic development of judoka, not only encouraging technical proficiency but also fostering intergenerational engagement and mutual learning within the sport [38,39,42,75,76,77].

### Limitations

Whilst both the reviews and the selected online courses were evaluated positively, some limitations need to be highlighted. First, the inclusion criterium of scientific contributions published in English did not allow for a comprehensive and globally relevant synthesis of evidence, which might have affected the validity of findings and the equity of scientific sources [16]. Second, this review included some studies not presenting an independent dual evaluation at all review phases and/or qualitative or quantitative assessments of heterogeneity [46]. Finally, the evaluation process of the online courses was limited to the educational programmes of the IJF Academy, not considering regional/national/continental resources, if present. Therefore, further research is necessary, as well as the collection of the practitioners’ experiences, perspectives, and challenges within intergenerational judo activities [42,49,78].

## 5. Conclusions

By summarising the findings of 16 systematic literature reviews and two international online judo courses in a framework, the present research provides novel and valuable insights into intergenerational judo, built upon a large sample size, wellbeing, and performance across various athletic levels, sex, and age. The framework encompasses techniques, moral code, roles, responsibilities, refereeing, safety, and age-specific belt examination requirements, as well as theoretical and applied modules on ageing, health, and organisation for young and older practitioners, highlighting the most relevant aspects of intergenerational judo programmes, such as aims, key characteristics, coach roles, barriers, and facilitators [20,25,26,34]. Transcending throws and pins, intergenerational judo weaves shared experiences, mutual respect, and lifelong learning. Therefore, on the tatami, individuals cradle wisdom, resilience, and the promise of a healthier, more connected society [1,9].

Future studies could complement existing evidence- and eminence-based knowledge by incorporating judo coaches’ perspectives through surveys or interviews [39,42,77], which would enable researchers to collect direct viewpoints and personal experiences, enhancing the comprehension of intergenerational dynamics, challenges, and advantages within the discipline. Finally, to provide valuable insights into the universality or variability of coaching philosophies and practices, the influence of culture- and setting-based factors should be investigated worldwide.

## Figures and Tables

**Table 2:** Information on judo practitioners, topics, and results reported in the included reviews.

Author (Year)	Judo Practitioners ^a^				Topic	Results
	Age (Years)	Sex (%)	Level	Sample		
Barreto et al. (2024) [50]	Adolescents to young adults	F = 100 M = 0	International	1485		Following each rule change (2010, 2013, 2015, 2017, and 2018), the CT changed towards homogeneity by weight divisions and increased Golden Score occurrence.
					Combat time (CT)	

Barreto et al. (2020) [51]	Adolescents to young adults	F = 0 M = 100	International	2562	Combat time (CT)	Following each rule change (2010, 2013, 2017, and 2018), the CT changed towards homogeneity by weight divisions and increased Golden Score occurrence.
Ciaccioni et al. (2019) [52]	Children to older people (55.0 ± 41.3)	F only = 38.2 M only = 20.6 Both = 32.4 NR = 8.8	Novice to international	1865	Bones	Positive association between judo and bone health/status emerged, with site-specific BMD accrual in judoka across the lifespan, bone turnover markers revealing a hypermetabolic status in high-level judo athletes, and fall techniques seemingly reducing bone impact force and velocity with respect to “natural” falls.
Gutierrez-Garcia et al. (2018) [53]	Children (5 to 10)	F = 20 M = 80	NR	602	Psychophysical effects	Young judoka showed improved fitness (arm bone density, flexibility, muscular endurance, agility) and reduced subcutaneous fat levels, similar to other sports, but also higher levels of anger than their peers.
Hlasho et al. (2023) [54]	NA	NA	NA	NA	Athlete success	Whilst volunteer-led federations seem inefficient and unsustainable for successfully planning an athlete’s international success pathway, professionalism and commercialization (e.g., financial resources, clear long-term plan, and full-time coaching and administration staff) appear central to improving athlete’s performance, participation, and efficacy in the athlete’s management systems.
Lakicevic et al. (2020) [55]	Young adults (20.5 ± 3.2)	NR	Competitive	1103	Rapid weight loss (RWL)	Inconsistent physiological data and biomarkers in athletes emerged, with psychological wellbeing parameters being more reliable. RWL increased tension, anger, and fatigue, while vigour decreased. The impact of RWL on performance was unclear. More research is needed to ensure athletes’ health, fairness, and sport benefits.
Lakicevic et al. (2024) [56]	Adolescents to young adults	F = 20 M = 80	Competitive	172	Rapid weight loss (RWL)	RWL leads to a significant rise in tension and a notable decrease in vigour. When judo athletes experience a weekly RWL of ≥5%, their mood states worsen significantly, regardless of gender.
Lockhart et al. (2022) [57]	Adults (24 ± NR; range: 18–65)	F = 6 M = 94	Novice to elite	158	Ukemi (breakfall techniques) and injury	Ukemi reduces kinematics compared to direct occipital contact, preventing head and neck injuries, with novice judoka showing larger hip, knee, and trunk flexion angles. A weak link exists between neck strength and improved ukemi, but fatigue negatively impacts breakfall skill.
Mooren et al. (2023) [58]	Adolescents to adults (range: 15–47)	NR	Competitive	361581	Injuries	Injury rates in judo tournaments vary, with 2.5–72.5% requiring medical evaluation and 1.1–4.1% causing game discontinuation. Common injury locations are the head, hand, knee, elbow, and shoulder, with sprains being the most frequent type, followed by contusions, skin lacerations, strains, and fractures. Injuries occur more often during standing fights.
Palumbo et al. (2023) [43]	Middle-aged and older people (63 ± 12)	F = 47 M = 53	Novice to expert	1392	Risks and benefits	On average, judo training later in life involves 2 ± 1 sessions per week, each lasting 61 ± 17 min, over a period of 7 ± 6 months. In the literature, health, functional fitness, and psychosocial aspects are key themes of judo training exposure and outcomes. Despite some methodological flaws, the current data suggest that judo training has positive effects as age advances.
Pečnikar et al. (2020) [59]	Children to adults	NR	Recreational to competitive	NR	Adapted judo	Increasingly used therapeutically, recreationally, and competitively, judo can be applied to people with special conditions (e.g., autism, ADHD, Down syndrome, intellectual and behavioural disorders), focusing on quality of life, motor skills, hyperactivity, health promotion, match analysis, and psychosocial effects. Low sample sizes and diverse research types limit the results’ generalisability.
Pocecco et al. (2013) [60]	Children to adults	NR	Competitive	NR	Injuries	11–12% injury risk (2008–2012 Olympic Games). Common: sprains, strains, and contusions (knee, shoulder, and fingers). Rare: brain and spine. Chronic: finger joints, lower back, and ears. Sex differences: inconsistent. Potential links: nutrition, hydration, weight cycling, psychological factors.
Rossi et al. (2022) [61]	Adolescents to adults	F = 34 M = 66	Regional to elite	850	Psychology of performance	↑ Tension, anger, anxiety, and nervousness in athletes facing defeat. ↓ Tension, anger, anxiety, and nervousness and ↑ motivation in athletes experiencing better performance.
Schoof et al. (2024) [62]	Adolescents to young adults	NR	Semi-elite to world-class elite	NR	Performance characteristics	Among the studied anthropometrical, physiological, technical, tactical, and psychological aspects, a broad set of physiological characteristics is needed to manage the demands of judo combats. Grip fighting-related characteristics discriminate between judoka of different performance levels.
Sterkowicz et al. (2019) [63]	Adolescents to young adults	M = 100	Novice to elite	724: 515 seniors & 209 juniors	Special Judo Fitness Test (SJFT)	Senior athletes (>21 years old) show a higher total number of throws and heart rate (HR) immediately after the SJFT, with limited differences for HR one minute after the SJFT between groups. Compared to juniors (<21 years old), more advanced athletes present a lower SJFT index and thus a better overall performance.
Sterkowicz et al. (2014) [64]	Adolescents to young adults	F = 100	Regional to international	161: 96 seniors & 65 juniors	Special Judo Fitness Test (SJFT)	According to the meta-analysis, the SJFT index shows a large effect size between ages, with seniors completing more throws than juniors. The smaller effect of HR immediately after and 1 min after the SJFT results in the throw number being a more significant factor in the differences between age categories.

Note: ^a^ Participants: age: when available, age (years) is reported as mean ± standard deviation values and range = min–max. Sex: F = females; M = males; ADHD = attention deficit hyperactivity disorder; BMD = bone mineral density; CT = combat time; HR = heart rate; NA = not applicable; NR = not reported; RWL = rapid weight loss; SJFT = Special Judo Fitness Test. ↑ = improvement/increase; ↓ decline/decrease.

**Table 3:** Characteristics and evaluation of international judo online courses by means of the NSQOL checklist tool.

Course	Aim and Typology	Modules	NSQOL Evaluation Criteria (Pt.) and Overall Rating							
			A	B	C	D	E	F	G	Rating
UCJI	Training instructors for youth and beginner athletes, combining theory and practical coaching, as well as judo techniques, fostering ongoing learning within and outside the IJF Academy - Mandatory	1. History of Judo 2. Classification of Judo—1 3. Culture of Judo 4. About IJF 5. Classification of Judo—2 6. Role of Instructor 7. Exercise Physiology I. 8. Classification of Judo—3 9. First Aid and Safety 10. Classification of Judo—4 11. LTAD Stages 12. Nage no Kata 13. Refereeing Rules 14. Practical Session	14	18	24	13	12	8	7	96/111 - Good
EdJCO	Empowering judo coaches with proper knowledge, skills, and attitudes for teaching and training older practitioners - Vocational	1. Organization and Environment 2. Aging Process 3. Safety and First Aid 4. Physiology and fitness 5. Psychology and Mental Health 6. Teaching and Training	15	16	19	11	12	7	7	87/111 - Good

EdJCO = Educating Judo Coaches for Older Practitioners; IJF = International Judo Federation; LTAD = long-term athlete development; UCJI = Undergraduate Certificate as Judo Instructor. Criteria (score range): A = Course Overview and Support (6–18 pt.); B = Content (6–18 pt.); C = Instructional Design (8–24 pt.); D = Learner Assessment (5–15 pt.); E = Accessibility and Usability (4–12 pt.); F = Technology (5–15 pt.); G = Course Evaluation (3–7 pt.).

## Data Availability

No new data were created.

## Supplementary Materials

The following supporting information can be downloaded at: https://www.mdpi.com/article/10.3390/sports12070177/s1, Supplementary Material S1. Methodological quality of the included review studies evaluated by means of the Quality Assessment Tool (National Institutes of Health, 2014). Supplementary Material S2. Quality assessment for online judo courses evaluated by means of the National Standards for Quality Online Learning (NSQOL) tool (Minnesota State Board of Assessors, 2024).
