# Supplementary material for: Intergenerational Judo: Synthesising Evidence- and Eminence-Based Knowledge on Judo across Ages
Source: Sports (Basel). 2024 Jun 26;12(7):177. doi: 10.3390/sports12070177 (PMC11281159; doi:10.3390/sports12070177)
Supplement: Supplementary file 1 [file sports-12-00177-s001.zip › Supplementary Material S1 - Study Quality Assessment.pdf]

Supplementary Material S1. Methodological quality of the included review studies evaluated by means of the Quality Assessment Tool (National Institutes of Health, 2014).

	Checklist Items for Observational Cohort and Cross-Sectional Studies[b]								
Author (year)	*1*	*2*	*3*	*4*	*5*	*6*	*7*	*8*	QRS[c]
**Barreto et al. (2024) [50]**	1	1	1	1	1	1	0	1	Excellent 7/8
**Barreto et al. (2022) [51]**	1	1	1	1	1	1	1	1	Excellent 8/8
**Ciaccioni et al. (2019) [52]**	1	1	1	1	1	1	0	1	Excellent 7/8
**Gutierrez-Garcia et al. (2018) [53]**	1	1	1	1	0	1	0	0	Good 5/8
**Hlasho et al. (2023) [54]**	0	1	1	1	1	0	0	0	Fair 4/8
**Lakicevic et al. (2020) [55]**	1	1	1	1	1	1	0	1	Excellent 7/8
**Lakicevic et al. (2024) [56]**	1	1	1	1	0	1	0	0	Good 5/8
**Lockhart et al. (2022) [57]**	1	1	1	1	1	1	0	1	Excellent 7/8
**Mooren et al. (2023) [58]**	1	1	1	1	1	1	1	1	Excellent 8/8
**Palumbo et al. (2023) [43]**	1	1	1	1	1	1	0	0	Good 6/8
**Pečnikar et al. (2020) [59]**	1	1	1	0	1	1	0	0	Good 5/8
**Pocecco et al. (2013) [60]**	1	1	1	0	0	0	0	0	Fair 3/8
**Rossi et al. (2022) [61]**	1	1	1	1	1	1	0	0	Good 6/8
**Schoof et al. (2024) [62]**	1	1	1	1	1	1	0	0	Good 6/8
**Sterkowicz et al. (2019) [63]**	1	1	1	0	0	1	0	1	Good 5/8
**Sterkowicz et al. (2014) [64]**	1	0	1	0	0	1	0	0	Fair 3/8

[a] see References.

[b] Scale items: 1. Focused question; 2. Eligibility criteria; 3. Literature search; 4. Dual review; 5. Internal validity; 6. Included studies description; 7. Publication bias; and 8. Heterogeneity. ''1'' indicates a ''yes'' score, and ''0'' indicates a ''no'' score or criterion not reported, or not determinable, or not applicable.

[c] Quality Rating Score: Excellent=7-8, Good=6-5, Fair=4-3, Poor=0-2.

References

43. Palumbo, F.; Ciaccioni, S.; Guidotti, F.; Forte, R.; Sacripanti, A.; Capranica, L.; Tessitore, A. Risks and Benefits of Judo Training for Middle-Aged and Older People: A Systematic Review. *Sports* **2023**, *11*, 68. https://doi.org/10.3390/sports11030068.
50. Barreto, L.B.M.; Duarte, T.S.; Ahmedov, F.; Aedo-munoz, E.A.; Martins, F.J.A.; Soto, D.A.S.; Miarka, B.; Brito, C.J. Combat Time in International Female Judo: A Systematic Review and Meta-Analysis. *Ido Mov. Cult.-J. Martial Arts Anthropol.* **2024**, *24*, 39–49. https://doi.org/10.14589/ido.24.2.5.
51. Barreto, L.B.M.; Santos, M.A.; Fernandes Da Costa, L.O.; Valenzuela, D.; Martins, F.J.; Slimani, M.; Bragazzi, N.L.; Miarka, B.; Brito, C.J. Combat Time in International Male Judo Competitions: A Systematic Review and Meta-Analysis. *Front. Psychol.* **2022**, *13*, 817210. https://doi.org/10.3389/fpsyg.2022.817210.
52. Ciaccioni, S.; Condello, G.; Guidotti, F.; Capranica, L. Effects of Judo Training on Bones: A Systematic Literature Review. *J. Strength. Cond. Res.* **2019**, *33*, 2882–2896. https://doi.org/10.1519/JSC.0000000000002340.
53. Gutierrez-Garcia, C.; Astrain, I.; Izquierdo, E.; Gomez-Alonso, M.T.; Yague, J.M. Effects of judo participation in children: A systematic review. *Ido Mov. Cult.-J. Martial Arts Anthropol.* **2018**, *18*, 63–73. https://doi.org/10.14589/ido.18.4.8.
54. Hlasho, T.S.; Thaane, T.; Mathunjwa, M.L.; Shaw, B.S.; Shandu, N.M.; Shaw, I. Past, Present and Future of Judo: A Systematic Review. *Heliyon* **2023**, http://dx.doi.org/10.2139/ssrn.4642402.

55. Lakicevic, N.; Roklicer, R.; Bianco, A.; Mani, D.; Paoli, A.; Trivic, T.; Ostojic, S.M.; Milovancev, A.; Maksimovic, N.; Drid, P. Effects of Rapid Weight Loss on Judo Athletes: A Systematic Review. *Nutrients* **2020**, *12*, 1220. https://doi.org/10.3390/nu12051220.
56. Lakicevic, N.; Thomas, E.; Isacco, L.; Tcymbal, A.; Pettersson, S.; Roklicer, R.; Tubic, T.; Paoli, A.; Bianco, A.; Drid, P. Rapid weight loss and mood states in judo athletes: A systematic review. *Eur. Rev. Appl. Psychol.-Rev. Eur. De Psychol. Appl.* **2024**, *74*, 100933. https://doi.org/10.1016/j.erap.2023.100933.
57. Lockhart, R.; Blach, W.; Angioi, M.; Ambrozy, T.; Rydzik, L.; Malliaropoulos, N. A Systematic Review on the Biomechanics of Breakfall Technique (Ukemi) in Relation to Injury in Judo within the Adult Judoka Population. *Int. J. Environ. Res. Public Health* **2022**, *19*, 4259. https://doi.org/10.3390/ijerph19074259.
58. Mooren, J.; von Gerhardt, A.L.; Hendriks, I.T.J.; Tol, J.L.; Koëter, S. Epidemiology of Injuries during Judo Tournaments. *Transl. Sports Med.* **2023**, *2023*, 2713614. https://doi.org/10.1155/2023/2713614.
59. Pečnikar Oblak, V.; Karpljuk, D.; Vodičar, J.; Simenko, J. Inclusion of people with intellectual disabilities in judo: A systematic review of literature. *Arch. Budo* **2020**, *16*, 245–260.
60. Pocecco, E.; Ruedl, G.; Stankovic, N.; Sterkowicz, S.; Del Vecchio, F.B.; Gutierrez-Garcia, C.; Rousseau, R.; Wolf, M.; Kopp, M.; Miarka, B.; et al. Injuries in judo: A systematic literature review including suggestions for prevention. *Br. J. Sports Med.* **2013**, *47*, 1139–1143. https://doi.org/10.1136/bjsports-2013-092886.
61. Rossi, C.; Roklicer, R.; Tubic, T.; Bianco, A.; Gentile, A.; Manojlovic, M.; Maksimovic, N.; Trivic, T.; Drid, P. The Role of Psychological Factors in Judo: A Systematic Review. *Int. J. Environ. Res. Public Health* **2022**, *19*, 2093. https://doi.org/10.3390/ijerph19042093.
62. Schoof, S.; Sliedrecht, F.; Elferink-Gemser, M.T. Throwing it out there: Grip on multidimensional performance characteristics of judoka—A systematic review. *Int. J. Sports Sci. Coach.* **2024**, *19*, 908–928. https://doi.org/10.1177/17479541231223160.
63. Sterkowicz-Przybycien, K.; Fukuda, D.H.; Franchini, E. Meta-Analysis to Determine Normative Values for the Special Judo Fitness Test in Male Athletes: 20+ Years of Sport-Specific Data and the Lasting Legacy of Stanislaw Sterkowicz. *Sports* **2019**, *7*, 194. https://doi.org/10.3390/sports7080194.
64. Sterkowicz-Przybycien, K.L.; Fukuda, D.H. Establishing normative data for the special judo fitness test in female athletes using systematic review and meta-analysis. *J. Strength Cond. Res.* **2014**, *28*, 3585–3593. https://doi.org/10.1519/JSC.0000000000000561.
