# Supplementary material for: Intergenerational Judo: Synthesising Evidence- and Eminence-Based Knowledge on Judo across Ages
Source: Sports (Basel). 2024 Jun 26;12(7):177. doi: 10.3390/sports12070177 (PMC11281159; doi:10.3390/sports12070177)
Supplement: Supplementary file 1 [file sports-12-00177-s001.zip › Supplementary Material S2 - Courses.pdf]

Supplementary Material S2. Quality assessment for online judo courses evaluated by means of the National Standards for Quality Online Learning (NSQOL) tool (Minnesota State Board of Assessors, 2024).

CHECKLIST FOR ONLINE COURSE DEVELOPMENT

This checklist has been adapted from the National Standards for Quality Online Learning, Third Edition, 2019. The Board recognizes that a checklist will not uniformly apply to all online courses; however, the checklist reflects Board expectations regarding the quality of online educational offerings that meet assessor certification or continuing education needs. Ideally, the checklist would be applied at a time when a course is being developed.

“Undergraduate Certificate as Judo Instructor” (UCJI) online course.

A: COURSE OVERVIEW AND SUPPORT

The overall design of the course is made clear to the learner at the beginning of the course. Course materials include support services essential to learner and instructor success. Course instructions articulate or link to relevant information and services.

A1. A course overview and syllabus are provided. 2

EXPLANATIONS

The course goals and objectives are measurable and clearly state what the participants will know or be able to do at the end of the course.

EXAMPLES

Even if the course goals are clearly state during the course, there isn’t a paragraph that explain that at the beginning of the course.

A2. Minimum computer skills, digital literacy skills, and technology requirements expected of the learner are clearly stated. 3

EXPLANATIONS

No particular or specific operating system or processor, speed, plug-in or connection are required, as well as no particular technology skills.

EXAMPLES

You can view courses on any devices that support these browsers. This includes mobile devices such as iPhones, iPads, and Android-enabled phones and tablets. You can also download all lessons in pdf format.

A3. If the course is instructed, biographical information and information on how to communicate with the instructor are provided to learners. 3

EXPLANATIONS

Learners can easily use a chat or discussion forum to communicate with instructors.

EXAMPLES

To communicate with the online teacher and course provider including information on the process for these communications, the students can use the specific chat and discussion forum: https://academy.ijf.org/forum/judo-instructor-level-1/history-of-judo_155322/1
Moreover, learners can use a chat in which they can choose any instructor of the course to start a conversation.

A4. Course expectations and policies applicable to learners are clearly stated and readily accessible within the introductory material of the course. 1

EXPLANATIONS

Privacy policies and course expectations are not always clearly stated.

EXAMPLES

All privacy policies are not clearly stated in the introductory material of the course.

A5. Grading policies and practices are clearly defined in accordance with course content learning expectations. 2

EXPLANATIONS

Grading rubrics and models of partially to fully completed assignments are not provided by the course.

EXAMPLES

The assessment process focuses on providing practice test for each lesson.

A6. The course provides a description or link to any orientation offered prior to the start of the course or any other technical support offered. 3

EXPLANATIONS

Sufficient learning resources and materials to increase student success are available to students before the course begins.

EXAMPLES

The course is easy to reach and use at this link:

https://academy.ijf.org/courses/instructor/lectures

B: CONTENT

The course promotes learner mastery of content.

B1. The course objectives or competencies are measurable and clearly state what the learner will be able to demonstrate as a result of successfully completing the course. 3

EXPLANATIONS

Objectives are matched to content requirements and to the grade and skill levels of the intended audience.

EXAMPLES

Students can clearly see how the content they engage with, the activities they complete, and the assessments they undertake are all designed to help them achieve the desired learning outcomes.

B2. The course expectations are consistent with course-level objectives or competencies, are representative of the structure of the course, and are clearly stated. 3

EXPLANATIONS

The course requirements for Judo coaches are in harmony with the course aims and objectives, accurately reflect the scope of the course content, and are articulated with clarity.

EXAMPLES

The course present clear and relevant information in a structured format, enabling students to comprehend, analyze, and synthesize contents.

B3. Any necessary digital literacy and communication skills are incorporated and taught as an integral part of the curriculum. 3

EXPLANATIONS

Promotes student-to-student communication as part of lesson activities.

EXAMPLES

Each slide of the online course present clear and relevant information in a structured format, enabling students to comprehend, analyze, and synthesize contents literacy is addressed. Learners can choose between pdf or ppt format to follow the lessons.

B4. Where needed, supplemental learning resources or related instructional materials are available to support and enrich learning and are aligned to the specific

content being delivered. 3

EXPLANATIONS

Orientations, tutorials, activities, policies and procedures to help students acquire the prerequisite knowledge and skills.

EXAMPLES

Instructor resources are included as additional guidance for teachers, possible scenarios for course module sequence and suggestions for guiding students on paths. Also, different documents correlating to practical lessons are available at the end of the course.

B5. The course content and supporting materials reflect a culturally diverse perspective that is free of bias. 3

EXPLANATIONS

Rich media are provided in multiple formats for ease of use and accessibility.

EXAMPLES

The course is available online and it is possible to download all course material.

B6. The course materials (e.g., textbooks or primary source documents) that support course content standards are accurate and current. 3

EXPLANATIONS

Images, language and content and selection of print and non-print resources are accurate.

EXAMPLES

This includes careful consideration of images, language, and the variety of print and non-print materials used in the learning activities.

C: INSTRUCTIONAL DESIGN

The course incorporates instructional materials, activities, resources, and assessments that engage all learners and support the achievement of learning goals.

C1. The course design includes activities that promote ownership of learning and self- monitoring. 3

EXPLANATIONS

The course structure includes adequate and appropriate methods and procedures to assess students' mastery of content.

EXAMPLES

The course include "weekly test" to provide automated, immediate feedback to students, including content that explains the error or alludes to the content.

C2. The course content and learning activities promote the achievement of the stated learning objectives or competencies. 3

EXPLANATIONS

The course provide a comprehensive course overview and syllabus.

EXAMPLES

Students have a clear understanding of the course structure, objectives and expectations from the beginning.

C3. The course is organized by units and lessons that fall into a logical sequence. 3

EXPLANATIONS

The course is organized by units/weeks and the associated lessons.

EXAMPLES

The course is organized in 11 units composed by different number of lessons (L1-L2-L3 etc.).

C4. The course content is appropriate to the intended learners. 3

EXPLANATIONS

Objectives are matched to content requirements and to the grade and skill levels of the intended audience.

EXAMPLES

Intended learners have a clear understanding of the course structure. Students benefit from free and online resources that enhance the course contents.

C5. The course design includes introductory assignments or activities that engage learners. 3

EXPLANATIONS

Several learning activities are performed during the course.

EXAMPLES

The course organized practical sessions for the learners.

C6. The course provides learners with multiple paths as appropriate, based on learner needs, to engage learners in a variety of ways. 3

EXPLANATIONS

The course includes guidelines for students on the use of copyrighted materials, appropriate use of networked resources and academic honesty.

EXAMPLES
Students engage with academic resources responsibly, ethically, and legally, fostering a culture of integrity and respect for intellectual property within the educational community.

C7. If the course is instructed, the course design provides opportunities for learner-instructor interaction, including opportunities for feedback about learner progress. 3

EXPLANATIONS

The course provides several opportunities for learner-instruction interaction.

EXAMPLES

All students can easily access to a chat to discuss with any instructor of the course.

C8. Course instructional materials and resources present content in an effective, engaging, and appropriate manner. 3

EXPLANATIONS

The course materials are carefully curated to align with the learning objectives and outcomes, ensuring that the content is relevant and meaningful to the students' academic and professional development.

EXAMPLES

The project is characterized by clarity structuring, ensuring originality and eliminating any possibility of plagiarism.

D: LEARNER ASSESSMENT

A variety of assessment strategies are used throughout the course, geared toward learning and engagement, and learners are provided with feedback on their progress.

D1. Clearly defined expectations for varied levels of proficiency are created and shared with learners. 2

EXPLANATIONS

The course doesn't always meet these criteria.

EXAMPLES

All expectations are communicated to the learners so they understand what is required of them at each proficiency level.

D2. Valid course assessments measure learner progress toward mastery of content. 3

EXPLANATIONS

This statement emphasizes the importance of assessments that accurately reflect student learning and provide meaningful feedback to both learners and instructors about their progress in mastering the course content.

EXAMPLES

The course include "weekly Test" and practice types of assignments to provide automated, immediate feedback to students, including content that explains the error or alludes to the content.

D3. Learner assessments are linked to the stated course, unit, or lesson-level objectives or competencies. 3

EXPLANATIONS

The course structure includes adequate and appropriate methods and procedures to assess students' mastery of content

EXAMPLES

A "weekly test" has been added at the conclusion of each lesson to reinforce learning procedures and assess students' comprehension.

D4. Assessment practices provide routine and varied opportunities for self-monitoring and reflection of learning. 3

EXPLANATIONS

Valid and reliable assessments are frequent and timely and include "self-check" and practice types of assignments to provide automated.

EXAMPLES

The course include “weekly test” and practice types of assignments to provide automated, immediate feedback to students, including content that explains the error or alludes to the content.

D5. Assessment materials provide the learner with the flexibility to demonstrate mastery in a variety of ways. 2

EXPLANATIONS

The course doesn’t meet always these criteria.

EXAMPLES

The assessment process focuses solely on providing feedback on correct and incorrect responses, without assigning grades. Other assessment available coulkd be provide during practical lesson of the course.

E: ACCESSIBILITY AND USABILITY

The course design reflects a commitment to accessibility, so that all learners can access all content and activities and easily navigate and interact with all course components. Course materials, activities, and assessments are designed to ensure that all learners have access to the same information and are able to engage in the same interactions within the same time frame.

E1. Course navigation is logical, consistent, and efficient from the learner's point of view. 3

EXPLANATIONS

The course is easy to navigate.

EXAMPLES

The course is easy to reach and use at this link:

https://academy.ijf.org/courses/instructor/lectures

E2. The course design facilitates readability. 3

EXPLANATIONS

Course design reflects a clear understanding of student needs and incorporates easy readability.

EXAMPLES

The entire course is customized for Judo coaches, integrating various teaching methods throughout the slides: Original documents, pictures, video.

E3. The course provides accessible course materials and activities to meet the needs of diverse learners. 3

EXPLANATIONS

Electronic resources meet national accessibility standards for publishers to ensure distribution of accessible, alternative versions of textbooks and other instructional materials.

EXAMPLES

Students benefit from free and online resources that enhance the course contents.

E4. Course multimedia facilitate ease of use. 3

EXPLANATIONS

No particular or specific technology skills are required to use this course.

EXAMPLES

The course includes multiple multimedia activities that actively engage students in

the learning process, particularly through the format of slides and video lessons.

F: TECHNOLOGY

The technologies enabling the various course components facilitate active learning and do not impede the learning process.

F1. Educational tools ensure learner privacy and confidentiality of learner information in accordance with any local, state, and national laws for learner data. 1

EXPLANATIONS

The course doesn't meet this criteria.

EXAMPLES

No local, state and national assessments are required, but the course content and assignments provide students with a rigorous and comprehensive learning experience. Local, state and national bodies can freely use the IJF course to develop the own courses including tasks and assessments.

F2. The course tools support the learning objectives or competencies. 2

EXPLANATIONS

Assessment strategies and tools make the student continuously aware of his/her progress in class.

EXAMPLES

Students can check and monitor scores of each Weekly Test.

F3. If the course is instructed, options are provided for the instructor to adapt activities that
accommodate learners' needs and preferences. 2

EXPLANATIONS

The course doesn't always meet these criteria.

EXAMPLES

The course requirements for Judo coaches are in harmony with the course aims and objectives, accurately reflect the scope of the course content, and are articulated with clarity.

F4. If the course is instructed, instructors control the release of content. 2

EXPLANATIONS

By controlling the release of content, instructors can maintain a coherent learning

experience for students, ensuring that they cover topics in a logical sequence and provide appropriate context or background information as needed.

EXAMPLES

The course architecture also permits the online teacher to add content, activities and assessments to extend learning opportunities.

F5. The course provides the necessary technical functionality to score and record assessments and calculate earned course points or grades. 1

EXPLANATIONS

The course doesn’t meet this criteria.

EXAMPLES

For the tests there aren’t grading rubrics or models provided by teachers.

G: COURSE EVALUATION

The course is evaluated regularly for effectiveness, using a variety of assessment strategies, and the findings are used as a basis for improvement. The course is kept up to date, both in content and in the application of new research on course design and technologies.

G1. The course uses multiple methods and sources of input for assessing course effectiveness. 3

EXPLANATIONS

State review of courses and accreditation of providers are ways to ensure that the course provider is of quality.

EXAMPLES

The course provider is authorized to operate on web pages where the course is offered.

G2. The course is reviewed to ensure that the content is current. 2

EXPLANATIONS

The course undergoes regular reviews to guarantee that the content remains up-to-date and relevant.

EXAMPLES

Students benefit from free and online resources that enhance the course contents.

G3. The course is updated on a continuous improvement cycle for effectiveness, based on the findings from ongoing reviews. 2

EXPLANATIONS

The course maintains its effectiveness through continuous improvement cycles, integrating insights from ongoing reviews to ensure relevance and quality.

EXAMPLES

Materials and/or Web links are always reviewed for appropriateness and are aligned with course objectives and specifications.

IJF

Section A: 14/18

Section B: 18/18

Section C: 24/24

Section D: 13/15

Section E: 12/12

Section F: 8/15

Section G: 7/9

CHECKLIST FOR ONLINE COURSE DEVELOPMENT

This checklist has been adapted from the National Standards for Quality Online Learning, Third Edition, 2019. The Board recognizes that a checklist will not uniformly apply to all online courses; however, the checklist reflects Board expectations regarding the quality of online educational offerings that meet assessor certification or continuing education needs. Ideally, the checklist would be applied at a time when a course is being developed.

“Educating Judo Coaches for Older practitioners” (EdJCO) online course.

A: COURSE OVERVIEW AND SUPPORT

The overall design of the course is made clear to the learner at the beginning of the course. Course materials include support services essential to learner and instructor success. Course instructions articulate or link to relevant information and services.

A1. A course overview and syllabus are provided. 3

EXPLANATIONS

The course goals and objectives are measurable and clearly state what the participants will know or be able to do at the end of the course.

EXAMPLES

‘Empowering judo coaches with proper knowledge, skills and attitudes for teaching and training older practitioners is the primary aim of the EdJCO project and online education program’.

A2. Minimum computer skills, digital literacy skills, and technology requirements expected of the learner are clearly stated. 3

EXPLANATIONS

No particular or specific operating system or processor, speed, plug-in or connection are required, as well as no particular technology skills.

EXAMPLES

You can view courses on any devices that support these browsers. This includes mobile devices such as iPhones, iPads, and Android-enabled phones and tablets.

A3. If the course is instructed, biographical information and information on how to communicate with the instructor are provided to learners. 2

EXPLANATIONS

The course is targeted for adult Judo coaches.

EXAMPLES

To communicate with the online teacher and course provider including information on the process for these communications, the students can use the specific form on the edjco.eu/contact/ or academy.ijf.org/contact web pages.

A4. Course expectations and policies applicable to learners are clearly stated and readily accessible within the introductory material of the course. 2

EXPLANATIONS

Privacy policies and course expectations are clearly stated.

EXAMPLES

All privacy policies are clearly stated both in in the course materials and in the orientation to the course materials.

A5. Grading policies and practices are clearly defined in accordance with course content learning expectations. 2

EXPLANATIONS

Grading rubrics and models of partially to fully completed assignments are not provided by the course.

EXAMPLES

The assessment process focuses solely on providing feedback on correct and incorrect responses.

A6. The course provides a description or link to any orientation offered prior to the start of the course or any other technical support offered. 3

EXPLANATIONS

Sufficient learning resources and materials to increase student success are available to students before the course begins.

EXAMPLES

The course is easy to reach and use at this link:

https://rise.articulate.com/share/jnRRZ1-chwwCMM6aUdo-GTY7caSif4_a#/

B: CONTENT

The course promotes learner mastery of content.

B1. The course objectives or competencies are measurable and clearly state what the learner will be able to demonstrate as a result of successfully completing the course. 3

EXPLANATIONS

Objectives are matched to content requirements and to the grade and skill levels of the intended audience.

EXAMPLES

Students can clearly see how the content they engage with, the activities they complete, and the assessments they undertake are all designed to help them achieve the desired learning outcomes.

B2. The course expectations are consistent with course-level objectives or competencies, are representative of the structure of the course, and are clearly stated. 3

EXPLANATIONS

The course requirements for Judo adult coaches are in harmony with the course aims and objectives, accurately reflect the scope of the course content, and are articulated with clarity.

EXAMPLES

The course present clear and relevant information in a structured format, enabling students to comprehend, analyze, and synthesize contents.

B3. Any necessary digital literacy and communication skills are incorporated and taught as an integral part of the curriculum. 2

EXPLANATIONS

Promotes student-to-student communication as part of lesson activities.

EXAMPLES

Each slide of the online course present clear and relevant information in a structured format, enabling students to comprehend, analyze, and synthesize contents literacy is addressed.

B4. Where needed, supplemental learning resources or related instructional materials are available to support and enrich learning and are aligned to the specific content being delivered. 2

EXPLANATIONS

Orientations, tutorials, activities, policies and procedures to help students acquire the

prerequisite knowledge and skills.

EXAMPLES

Instructor resources are included as additional guidance for teachers, possible scenarios for course module sequence and suggestions for guiding students on paths. Different scientific papers from this project and course are available.

B5. The course content and supporting materials reflect a culturally diverse perspective that is free of bias. 3

EXPLANATIONS

Rich media are provided in multiple formats for ease of use and accessibility.

EXAMPLES

The course is available online and in e-book format.

B6. The course materials (e.g., textbooks or primary source documents) that support course content standards are accurate and current. 3

EXPLANATIONS

Images, language and content and selection of print and non-print resources are accurate.

EXAMPLES

This includes careful consideration of images, language, and the variety of print and non-print materials used in the learning activities.

C: INSTRUCTIONAL DESIGN

The course incorporates instructional materials, activities, resources, and assessments that engage all learners and support the achievement of learning goals.

C1. The course design includes activities that promote ownership of learning and self- monitoring. 3

EXPLANATIONS

The course structure includes adequate and appropriate methods and procedures to assess students' mastery of content.

EXAMPLES

The course include "self-check" and practice types of assignments to provide automated, immediate feedback to students, including content that explains the error or alludes to the content. They always have a second chance to repeat the self-evaluation.

C2. The course content and learning activities promote the achievement of the stated learning objectives or competencies. 3

EXPLANATIONS

The course excels in meeting this criteria by providing a comprehensive course overview and syllabus.

EXAMPLES

Students have a clear understanding of the course structure, objectives and expectations from the beginning.

C3. The course is organized by units and lessons that fall into a logical sequence. 3

EXPLANATIONS

The course is organized by units and the associated lessons.

EXAMPLES

The course is organised in 6 units/lessons composed by different number of chapters.

C4. The course content is appropriate to the intended learners. 3

EXPLANATIONS

Objectives are matched to content requirements and to the grade and skill levels of the intended audience.

EXAMPLES

Intended learners have a clear understanding of the course structure. Students benefit from free and online resources that enhance the course contents.

C5. The course design includes introductory assignments or activities that engage learners. 1

EXPLANATIONS

The course doesn't always meet these criteria.

EXAMPLES

Introductory assignments and activities enganged learners in previous meetings and congferences.

C6. The course provides learners with multiple paths as appropriate, based on learner needs, to engage learners in a variety of ways. 2

EXPLANATIONS

The course includes guidelines for students on the use of copyrighted materials, appropriate use of networked resources and academic honesty.

EXAMPLES

Students engage with academic resources responsibly, ethically, and legally, fostering

a culture of integrity and respect for intellectual property within the educational community.

C7. If the course is instructed, the course design provides opportunities for learner-instructor interaction, including opportunities for feedback about learner progress. 1

EXPLANATIONS

The course doesn't always meet these criteria.

EXAMPLES

The course doesn't provide student interaction but the project of EdJCO provided that in other meetings and previous occasions. All students interaction were provided suring previous meetings and conferences, where students had the opportunity to discuss about the course with the creators.

C8. Course instructional materials and resources present content in an effective, engaging, and appropriate manner. 3

EXPLANATIONS

The course materials are carefully curated to align with the learning objectives and outcomes, ensuring that the content is relevant and meaningful to the students' academic and professional development.

EXAMPLES

The project is characterized by clarity structuring, ensuring originality and eliminating any possibility of plagiarism. Each known scientific evidence is meticulously accompanied by a well-documented reference.

D: LEARNER ASSESSMENT

A variety of assessment strategies are used throughout the course, geared toward learning and engagement, and learners are provided with feedback on their progress.

D1. Clearly defined expectations for varied levels of proficiency are created and shared with learners. 2

EXPLANATIONS

The course doesn't always meet these criteria.

EXAMPLES

All expectations are communicated to the learners so they understand what is

required of them at each proficiency level.

D2. Valid course assessments measure learner progress toward mastery of content. 2

EXPLANATIONS

This statement emphasizes the importance of assessments that accurately reflect student learning and provide meaningful feedback to both learners and instructors about their progress in mastering the course content.

EXAMPLES

The course include "self-check" and practice types of assignments to provide automated, immediate feedback to students, including content that explains the error or alludes to the content. They always have a second chance to repeat the self-evaluation.

D3. Learner assessments are linked to the stated course, unit, or lesson-level objectives or competencies. 3

EXPLANATIONS

The course structure includes adequate and appropriate methods and procedures to assess students' mastery of content

EXAMPLES

A self-evaluation quiz has been added at the conclusion of each lesson to reinforce learning procedures and assess students' comprehension.

D4. Assessment practices provide routine and varied opportunities for self-monitoring and reflection of learning. 3

EXPLANATIONS

Valid and reliable assessments are frequent and timely and include "self-check" and practice types of assignments to provide automated.

EXAMPLES

The course include "self-check" and practice types of assignments to provide automated, immediate feedback to students, including content that explains the error or alludes to the content. They always have a second chance to repeat the self-evaluation

D5. Assessment materials provide the learner with the flexibility to demonstrate mastery in a variety of ways. 1

EXPLANATIONS

The course doesn't meet this criteria.

EXAMPLES

The The assessment process focuses solely on providing feedback on correct and incorrect responses, without assigning grades. There are not alternative evaluation methods available.

E: ACCESSIBILITY AND USABILITY

The course design reflects a commitment to accessibility, so that all learners can access all content and activities and easily navigate and interact with all course components. Course materials, activities, and assessments are designed to ensure that all learners have access to the same information and are able to engage in the same interactions within the same time frame.

E1. Course navigation is logical, consistent, and efficient from the learner's point of view. 3

EXPLANATIONS

The course is easy to navigate.

EXAMPLES

The course is easy to reach and use at this link:

https://rise.articulate.com/share/jnRRZ1-chwwCMM6aUdo-GTY7caSif4_a#/

E2. The course design facilitates readability. 3

EXPLANATIONS

Course design reflects a clear understanding of student needs and incorporates easy readability.

EXAMPLES

The entire course is customized for Judo coaches, integrating various teaching methods throughout the slides: Original documents, pictures, video and references.

E3. The course provides accessible course materials and activities to meet the needs of diverse learners. 3

EXPLANATIONS

Electronic resources meet national accessibility standards for publishers to ensure distribution of accessible, alternative versions of textbooks and other instructional materials.

EXAMPLES

Students benefit from free and online resources that enhance the course contents. The E-book meets national accessibility standards for publishers.

E4. Course multimedia facilitate ease of use. 3

EXPLANATIONS

No particular or specific technology skills are required to use this course.

EXAMPLES

The course includes multiple multimedia activities that actively engage students in the learning process, particularly through the format of slides and video lessons.

F: TECHNOLOGY

The technologies enabling the various course components facilitate active learning and do not impede the learning process.

F1. Educational tools ensure learner privacy and confidentiality of learner information in accordance with any local, state, and national laws for learner data. 1

EXPLANATIONS

The course doesn't meet this criteria.

EXAMPLES

No local, state and national assessments are required, bu the course content and assignments provide students with a rigorous and comprehensive learning experience. Local, state and national bodies can freely use the EdJCO course to develop the own courses including tasks and assessments.

F2. The course tools support the learning objectives or competencies. 2

EXPLANATIONS

Assessment strategies and tools make the student continuously aware of his/her progress in class.

EXAMPLES

Students can check and monitor scores of each Self-Evaluation Quiz.

F3. If the course is instructed, options are provided for the instructor to adapt activities that
accommodate learners' needs and preferences. 1

EXPLANATIONS

The course doesn't always meet these criteria.

EXAMPLES

The course requirements for Judo adult coaches are in harmony with the course aims and objectives, accurately reflect the scope of the course content, and are articulated with clarity.

F4. If the course is instructed, instructors control the release of content. 2

EXPLANATIONS

By controlling the release of content, instructors can maintain a coherent learning

experience for students, ensuring that they cover topics in a logical sequence and provide appropriate context or background information as needed.

EXAMPLES

The course architecture also permits the online teacher to add content, activities and assessments to extend learning opportunities.

F5. The course provides the necessary technical functionality to score and record assessments and calculate earned course points or grades. 1

EXPLANATIONS

The course doesn't meet this criteria.

EXAMPLES

For the tests there aren't grading rubrics or models provided by teachers.

G: COURSE EVALUATION

The course is evaluated regularly for effectiveness, using a variety of assessment strategies, and the findings are used as a basis for improvement. The course is kept up to date, both in content and in the application of new research on course design and technologies.

G1. The course uses multiple methods and sources of input for assessing course effectiveness. 3

EXPLANATIONS

State review of courses and accreditation of providers are ways to ensure that the course provider is of quality.

EXAMPLES

The course provider is authorized to operate on web pages where the course is offered.

G2. The course is reviewed to ensure that the content is current. 2

EXPLANATIONS

The course undergoes regular reviews to guarantee that the content remains up-to-date and relevant.

EXAMPLES

Students benefit from free and online resources that enhance the course contents.

G3. The course is updated on a continuous improvement cycle for effectiveness, based on the findings from ongoing reviews. 2

EXPLANATIONS

The course maintains its effectiveness through continuous improvement cycles, integrating insights from ongoing reviews to ensure relevance and quality.

EXAMPLES

Materials and/or Web links are always reviewed for appropriateness and are aligned with course objectives and specifications.

EdJCO

Section A: 15/18

Section B: 15/18

Section C: 19/24

Section D: 11/15

Section E: 12/12

Section F: 7/15

Section G: 7/9

111/185= poor

186/259= fair

260/333= good
